# Diagnostic Usefulness of Antibodies Against M-type Phospholipase A2 Receptor (aPLA2R) in Primary Membranous Nephropathy in the Mexican Population

**DOI:** 10.7759/cureus.43588

**Published:** 2023-08-16

**Authors:** Juan Daniel Diaz Garcia

**Affiliations:** 1 Nephrology, Centro Médico Nacional 20 de Noviembre, Mexico, MEX

**Keywords:** kidney biopsy, proteinuria, nephrotic syndrome, primary membranous nephropathy, apla2r

## Abstract

Objective: This study aimed to determine the frequency and diagnostic performance of antibodies against M-type phospholipase A2 receptors (aPLA2R) in subjects with idiopathic membranous nephropathy (IMN).

Materials and methods: A diagnostic test study was conducted in a cohort of 160 patients from the nephrology outpatient clinic over a period of eight years. Serum samples were taken and analyzed from patients with a histological diagnosis of IMN with proteinuria greater than and less than 1 g in 24 hours and other glomerular diseases other than IMN with aPLA2R measurement by enzyme-linked immunosorbent assay (ELISA) (Euroimmun, Luebeck, Germany).

Results: In 22 of 160 patients, an aPLA2R concentration >9 RU/mL was found, and all these cases had IMN. The prevalence of seropositivity in cases with active IMN was 78% (21/27). All these correlations were statistically significant with a p<0.001. The area under the receiver operating characteristic (ROC) curve (AB-ROC) of aPLA2R was 0.87 (95%, CI: 78-0.96).

Conclusions: The aPLA2R has adequate diagnostic usefulness to diagnose IMN in the selected population, especially in subjects with proteinuria greater than 1 gr/day, with a sensitivity of 78% and a specificity of 99%.

## Introduction

Idiopathic membranous nephropathy (IMN) is one of the main causes of nephrotic syndrome in the adult population [1]. In the Caucasian population, approximately 20-40% of patients progress to end-stage kidney disease in the 10 to 15 years following diagnostic [2]. The etiology and nature of the antigens that cause IMN are unknown. Some hypotheses suggest that the deposits come from circulating immunocomplexes, while others suggest in situ formation by antibodies that recognize either native or foreign antigens deposited in the glomerulus [3]. The disease was originally described by David Jones in 1957; he used silver methenamine stain (now called Jones’ methenamine stain) for his description and found basal membrane thickening with structural changes that resembled spikes, a characteristic finding in this disease [4].

In 2009, Beck et al. identified the M-type phospholipase A2 receptor (PLA2R) as an antigenic target in IMN which is found in the podocyte membrane, a key antigen for the autoimmune response in IMN [5]. They were discovered using Western blotting by mixing human glomeruli extract with sera of patients with IMN, secondary membranous nephropathy, other proteinuric nephropathies, and healthy kidneys. The last three groups were used as controls. A glycoprotein of 185 kD was identified in 70% of samples of patients with IMN but in none of the control groups. This glycoprotein was PLA2R which was identified by the antibodies union formed against its receptor (aPLA2R [ELISA; enzyme-linked immunosorbent assay], Euroimmun, Luebeck, Germany). From the moment of its discovery, many studies have demonstrated that disease activity is associated with the presence of aPLA2R (ELISA) in plasma, and the lowering of its levels corresponds with treatment response [6].

The description of these antibodies has generated great expectations about their usefulness to identify subgroups of IMN with different clinical profiles and prognoses and the possibility to differentiate between idiopathic and secondary disease forms [7-8]. Recognizing PLA2R as an antigenic target in IMN has led to trials designed to determine serum levels of aPLA2R (ELISA) as well as measure them in kidney tissue to diagnose IMN caused by aPLA2R. These trials have demonstrated a sensitivity of 70-82% and a specificity of 89-100% [9].

Currently, there are many trials from around the world (China, Europe, Iran) that show a frequency of IMN related to antibodies against aPLA2R (ELISA) between 69% and 82% [10].

As stated above, apart from Western blotting, other technics for aPLA2R determination can be used such as immunofluorescence and ELISA [11]. ELISA is the easiest and most inexpensive method, available in most clinical laboratories and hospitals. Hoxha et al. found an association between higher aPLA2R serum levels (measured by ELISA) and higher proteinuria in patients with IMN. During prospective follow-up, the reduction of aPLA2R (ELISA) serum levels was associated with a good response to immunosuppressant treatment [12].

The same authors that originally described the antibodies evaluated ELISA for their detection in 109 patients with IMN and observed that this essay was just as sensitive and specific as Western blotting [13]. Currently, evidence that associates IMN with aPLA2R (ELISA) has been demonstrated in multiple trials, especially in active forms of diseases [14-15]; however, the specificity of these tests has only been partially studied, and there have even been reports of positive results in subjects with secondary forms of membranous nephropathy [16-17].

## Materials and methods

This study was approved by the Research and Ethics Committee of the Institutional Review Board (IRB) with approval number 291.2021.

A cross-sectional diagnostic test trial was performed which consisted of a cross-sectional and observational phase in which blood samples were drawn from patients with histological diagnostic of IMN (with or without nephrotic syndrome) in search of the prevalence of aPLA2R (ELISA) positivity in a Mexican population with IMN. During the same time period, blood from patients with glomerular diseases other than INM like lupus nephritis, diabetic nephropathy, primary glomerular diseases other than INM, and kidney transplant donors (healthy subjects) searching for aPLA2R titers in populations different than IMN to evaluate tests specificity. All patients were recruited from the outpatient nephrology clinic.

Sample size

All patients with the diagnosis of IMN in follow-up by the outpatient nephrology clinic were included. Since the frequency of aPLA2R (ELISA) (prevalence) in our populations was the main outcome, we estimated it would make up approximately 75% based on precedent.

The following formula was used to calculate sample size for a proportion, with 95% confidence and 10% alpha error:

[Formula #1] n = (Z2 × P(1 - P))/e2

Where n is a simple size; Z is 1.96, which corresponds to a confidence value of 95% with an alpha error of 5%; P is the expected proportion of 75%; and e is the desired precision also known as an acceptable error of 10% (±0.1). Substituting from the formula, 73 IMN cases would be needed.

The formula can be adjusted to finite populations using formula #2:

[Formula #2] n(adj) = (Nxn)/(N+n)

Where n represents the sample size and N is the population with IMN. Therefore, substituting n=73 and N=90 in the formula, the final sample of subjects with IMN was 41.

Description of the method used for the detection of aPLA2R

This method is a semiquantitative or quantitative in vitro assay for the detection of PLA2R through IgG human antibodies from either sera or plasma. It consists of four stages: During the first stage, the patients’ sample is diluted, and the standard and positive and negative controls were incubated in pods covered in PLA2R. In the second stage, to detect antibody unions, IgG antibodies marked with an enzyme (enzymatic conjugate), which would also catalyze a color reaction, were incubated. For this to happen, the enzyme substrate was incubated again during the third stage. The last stage was halted with a stop solution (HCl), and a photometric measurement of the color intensity at 450 nm during the 30 min after adding the stop solution was taken.

Patients' sample preparation and stability

A 5 mL blood sample was taken from the patients’ forearm and placed in a blood collection tube without anticoagulant. The clot was allowed to retract for 30 minutes.

The sample was centrifuged for 20 minutes at 3500 rpm at a refrigeration temperature between 5 and 10 degrees Celsius.

The supernatant was aliquoted into three Eppendorf tubes, with an approximate volume of 0.7 ml each, and frozen in a -40* Celsius deep freezer until processing.

Euroinmmun anti-PLA2R (ELISA)

The linearity of this technique was in the concentration range of 6 RU/ml to 1500 RU/ml with an R2> 0.95 and a minimum detection of 0.6 RU/ml. The sensitivity was 97.5% with a specificity of 100%.

The reference interval for the median concentration of aPLA2R is 0.4 RU/mL with a range from 0.0 to 5.0 Ru/mL and a cutoff value of 20 RU/ml. The essay consisted of negative and positive internal controls with previously established reference values which gave direct evaluation to control samples.

Study population

The inclusion criteria included patients over 18 years of age treated in the nephrology or mineral metabolism outpatient clinics with a biopsy confirming the diagnosis of IMN and lupus nephritis, among others, and signed informed consent by the patient.

The exclusion criteria included patients with incomplete information (biopsy diagnosis not made by a nephropathologist or inability to analyze biopsy in the institute).

The elimination criteria included patients who withdrew their consent and patients with incomplete information.

Statistical analysis strategy

Descriptive statistics with measures of central tendency and dispersion of the data according to their distribution were used. The distribution of variables was analyzed using Kolmogorov-Smirnov Z. Frequencies and percentages were used for categorical variables. Median and interquartile ranges were used since most of the variables had a non-Gaussian distribution. ROC curve analysis of the aPLA2R (ELISA) values was performed to assess the diagnostic performance relative to the diagnosis of IMN in diagnostic entities. To analyze the groups with the presence of aPLA2R (ELISA) and without antibodies, the cut-off point with the best diagnostic performance was sought in the ROC curves. Comparisons between groups were made using the chi-square test in the case of proportions and the Mann-Whitney U test for continuous variables. The correlation between aPLA2R (ELISA) values and proteinuria or glomerular filtration rate was assessed using the Pearson or Spearman correlation coefficient depending on the distribution of the variables. The statistical analysis was carried out with SPSS Statistics version 19.0 (IBM Corp. Released 2010. IBM SPSS Statistics for Windows, Version 19.0. Armonk, NY: IBM Corp.) and the graphs with GraphPad Prism 5 (San Diego, California, USA.). Two-tailed p<0.05 was considered significant.

Recruitment and patient sign-up

Patients were recruited and invited to participate in the outpatient nephrology clinic, and those who accepted were handed out informed consent and given a subsequent appointment to talk over questions regarding the informed consent. Given the case, signatures were collected, and blood samples were drawn.

Data recollection and handling

For data capture, a computer application was used to input all the data into a database designed especially for the study. All the input was secured. A printout was designed to facilitate data recollection and to register each of the participants’ visits. All data was introduced as soon as possible after completing the evaluation. The physicians in charge logged personal data (full name and register number) into the database. After signing up, each patient was given an identification number. All information was handled in accordance with international data protection regulations.

## Results

In total, 106 subjects were included, 41 (39%) with IMN, 10 (9%) with pure class V lupus nephropathy, 21 (20%) with mixed lupus nephropathy, 16 (15%) with other primary glomerular disease (focal segmental glomerulosclerosis, membranoproliferative glomerulonephritis, IgA nephropathy, pauci-immune glomerulonephritis), 7 (7%) diabetic nephropathy, and 11% (10%) healthy donors.

The demographic variables of the subjects at the moment of the aPLA2R (ELISA) determination are summarized in Table 1. Subjects with nephropathy were older than other groups, with subjects with diabetic nephropathy representing the oldest among this category, both being statistically significant (p<0.01). Of the subjects with IMN diagnostic, 27 (66%) had proteinuria >1 gr/day. The median age in this group was 53 years (interquartile range [IR] of 39.7-66). No differences between the baseline characteristics in subjects with IMN with proteinuria <1 gr/day were observed.

**Table 1:** Baseline characteristic *Qualitative variables are expressed in mean and interquartile ranges. IR: interquartile range, GFR: glomerular filtration rate calculated with CKD-EPI

Variable*	IMN and proteinuria ≥ 1g/day	IMN and proteinuria <1 g/day	Lupus	Diabetes	Others
Female, n (%)	8 (30)	5 (36)	28 (90)	1 (14)	8 (50)
Male, n (%)	19 (70)	9 (64)	3 (10)	6 (86)	8 (50)
Age in years (IR*)	53 (39.7-66.2)	54 (47-5-60.5)	25 (22.7-28.2)	50 (41.5-56.2)	49 (37-52)
Proteinuria, gr/24hrs (IR)	6.7 (4.5-8.9)	0.4 (0.30-0.77)	3.4 (0.45-4.7)	2 (0.4-2.5)	2.5 (1.8-6.4)
GFR**, mL/min/1.73m^2^ (IR)	77 (72-107)	76 (64-94.5)	82 (45-115)	29 (14.2-57.5)	68 (40-95)
Cholesterol, mg/dl (IR)	225 (191-301)	174 (133-224.7)	223 (153-274)	244 (153.5-287)	270 (166-298)
Albumin, gr/L (IR)	3.0 (2.3-3.5)	4.2 (4.0-4.5)	3.4 (2.6-3.7)	3.5 (2.6-4.0)	3.1 (2.5-4.0)

**Figure 1:**
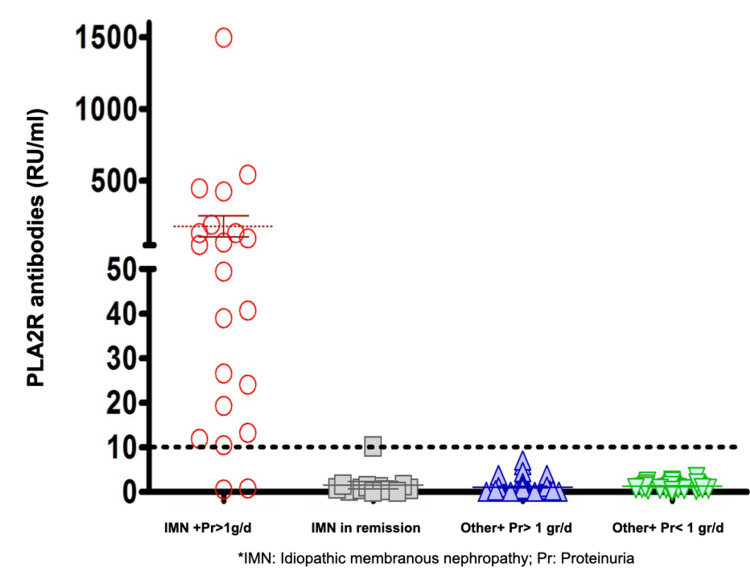
aPLA2R (ELISA) in different glomerular diseases PLA2R: M-type phospholipase A2 receptor, IMN: idiopathic membranous nephropathy, Pr: proteinuria

**Figure 2:**
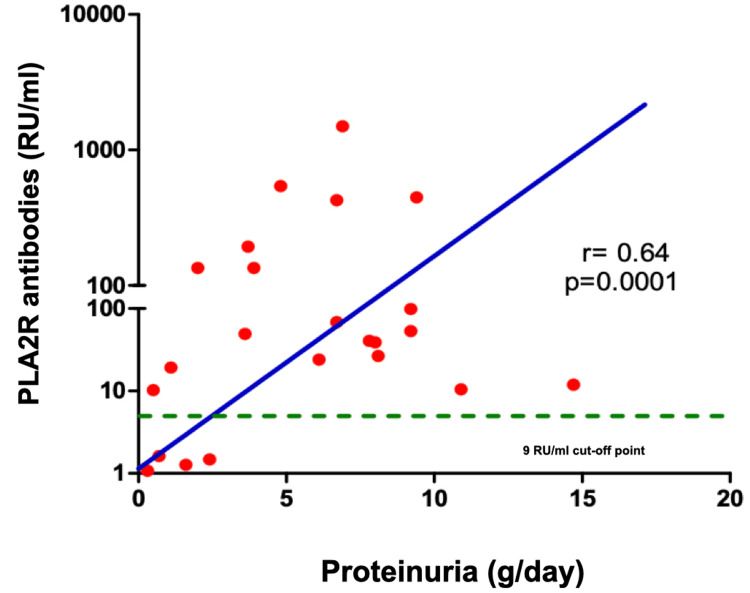
Correlation between aPLA2R (ELISA) titers and 24-hour proteinuria in IMN PLA2R: M-type phospholipase A2 receptors

An aPLA2R (ELISA) concentration >9 RU/ml was found in 22 of the 106 patients, all of which had IMN diagnosis (21 active IMN and 1 inactive case) (Figure 1). The prevalence of seropositivity for IMN cases with proteinuria above 1 gr/day was 78% (21/27). aPLA2R (ELISA) concentrations had a positive correlation with proteinuria in 24-hour urine (r=0.68, figure 2) and serum cholesterol (0.42) and negative correlation with serum albumin (-0.34). All these correlations were statistically significant with p<0.001.

**Figure 3:**
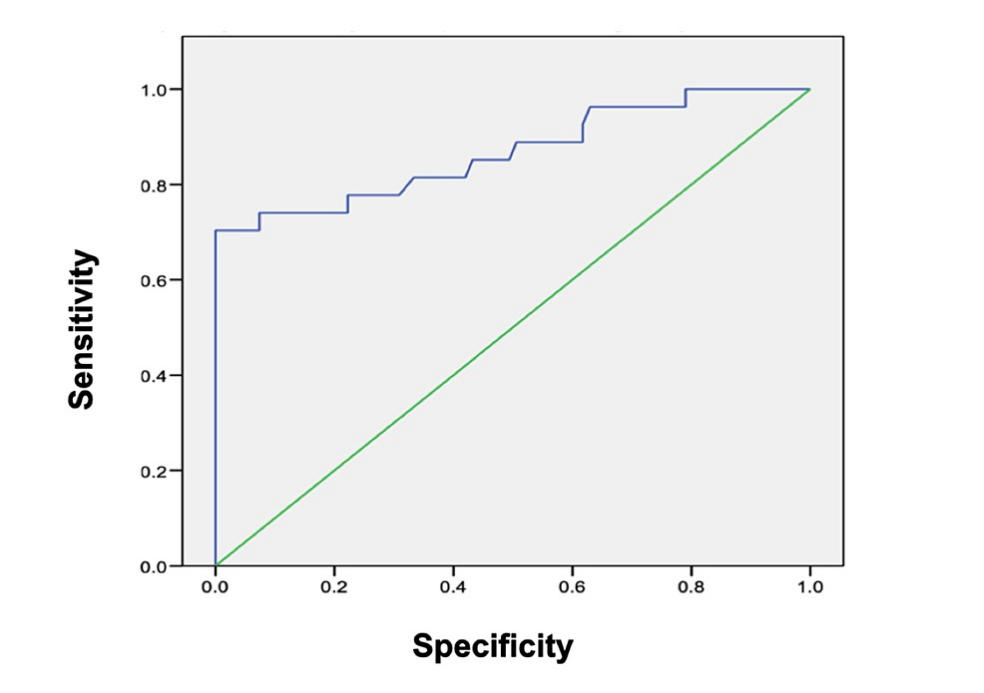
ROC curve comparing IMN diagnosis (including mesangial deposits) with other glomerular diseases

**Figure 4:**
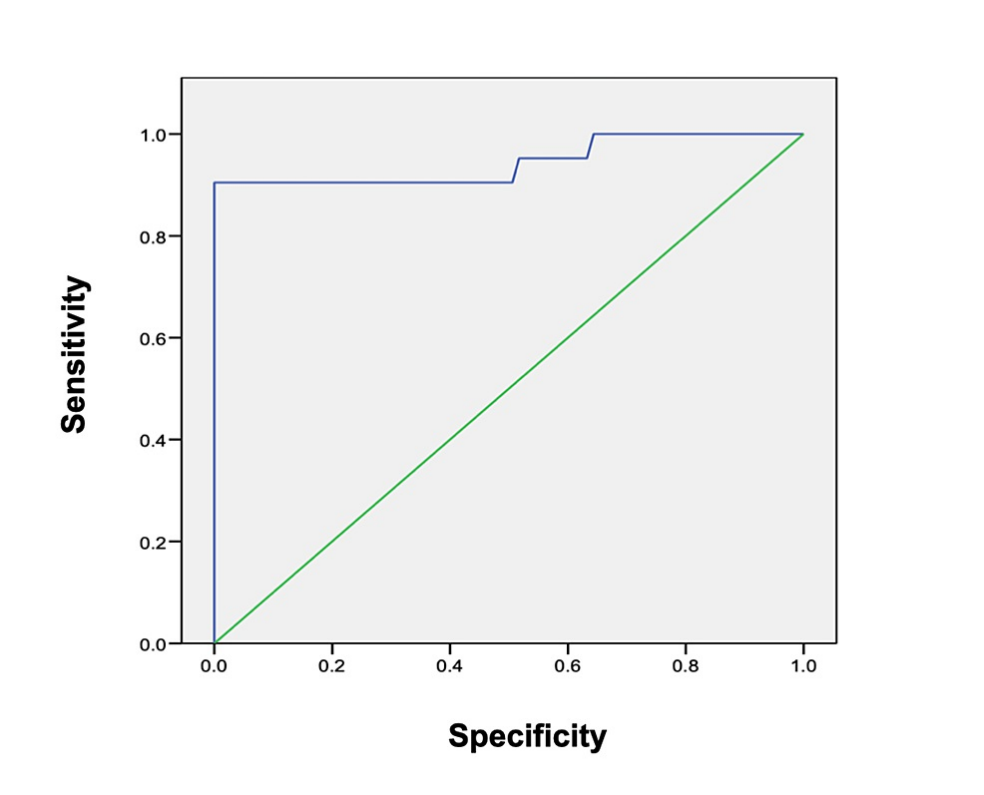
ROC curve comparing IMN diagnosis (excluding mesangial deposits) with other glomerular diseases

All subjects with active IMN and mesangial deposits (four patients) were negative for aPLA2R (ELISA). In these cases, even with histological diagnostic, no secondary cause was found during diagnostic approach. Of the subjects with IMN and proteinuria >1 gr/day, 21/27 had anti-PLA2R>9 RU/ml. The area under the ROC curve for aPLA2R (ELISA) was 0.87 (95%, CI;78-0.96) (Figure 3). When comparing subjects with IMN and proteinuria >1 gr/day and other subjects with glomerular disease with a cut-off point of 9 RU/ml, the sensibility was 78% and the specificity was 99%. If we exclude subjects with membranous nephropathy and mesangial deposits, the area under the ROC curve was 0.95 (95%. CI: 0.87-1.00). In this analysis, a cut-off point of 9 RU/ml has a sensibility of 91% and specificity of 99% (Figure 4).

## Discussion

The results of this study show that aPLA2R (ELISA) can be useful when diagnosing IMN in select populations, especially in subjects with proteinuria >1 gr/day. The frequency of aPLA2R (ELISA) found was similar to that reported in other series of patients with IMN and significant proteinuria. Testing for anti-PLA2R with ELISA in peripheral blood has a 78% sensitivity and 99% specificity. When excluding subjects whose kidney biopsy presents mesangial expansion, a histopathological sign of secondary membranous nephropathy, sensitivity rises to 91%. In these cases, even when the histopathological diagnostic suggests a secondary cause, it was not possible to find the etiology which suggests that membranous nephropathy with mesangial proliferation has a different autoimmune physiopathology with antibodies and clinical presentation different to that reported in IMN with anti-PLA2R. Other studies have found PLA2R in hepatitis B virus, generalized lupus erythematosus, and malignant neoplasm lowering its specificity [13]; however, the method used was Western blotting, which has a higher sensibility than ELISA augmenting the rate of false positive. Our study had a cut-off point of 9 RU/ml using ELISA finding a very high specificity.

Subjects with a previous IMN diagnostic who were clinically in remission with proteinuria under 1 gr/day had aPLA2R (ELISA) titers <9 RU/ml, most with values <1 RU/ml with the exception of one subject with titers of 10 RU/ml.

The correlation between antibodies and proteinuria wasn’t 100% which can be explained by the cross-sectional nature of the study. It’s probable that many patients with imminent relapse or early remission will present changes in aPLA2R (ELISA) earlier in comparison to proteinuria, a fact which was described in a patient’s titers who had undergone a renal transplant and presented IMN recurrence in his graft [9]. Other series have confirmed that aPLA2R (ELISA) rises before the presentation of proteinuria in relapses.

To our knowledge, this is the first study in the Latin-American population that shows that the prevalence of antibodies in IMN is similar to that reported in other populations [8-14]. Compared to other publications, antibodies were validated against a significant number of cases of immune diseases and a high prevalence of autoantibodies like generalized lupus erythematosus. The data presented shows the usefulness of a cut-off point of 9 RU/ml, finding no false positives.

This study should show the possibility of using ELISA to detect aPLA2R in the serum of patients with suspected glomerular diseases, especially those presenting with nephrotic syndrome without deterioration of glomerular filtration rate and proteinuria >1 gr/day. A value >9 RU/ml could be sufficient to diagnose IMN and avoid the need for a biopsy. Given the accumulated evidence, it is probable that a percentage of the so-called IMN should be renamed membranous nephropathy mediated by aPLA2R (ELISA), reserving the name idiopathic for those without the presence of these antibodies.

The limitations of the study are that due to its design, causality between the aPLA2R (ELISA) and the disease cannot be established. However, as it is a diagnostic test study, we believe that we have a highly specific test that is useful to the clinician. Another limitation secondary to the design was that the measurement of aPLA2R (ELISA) was not performed concomitantly at the time of the renal biopsy in all the patients, and there are no repeated aPLA2R (ELISA) measurements; therefore, the relationship between proteinuria and the behavior of aPLA2R (ELISA) cannot be adequately established.

## Conclusions

In conclusion, according to the results obtained in our study, aPLA2R (ELISA) is useful for the diagnosis of IMN, especially with proteinuria greater than 1g in 24 hours. Likewise, this study showed that although the best cut-off point for the ELISA method to detect anti-PLA2R antibodies in patients with IMN is lower (2-2.7 RU/ml) than that indicated by the manufacturer, our cut-off point agreed. Based on what was obtained, it guarantees a better diagnostic yield, standing at 9 RU/ml, so that titers above this cut-off point (9 RU/ml) exclude other glomerular diseases and are highly specific for the diagnosis of IMN. However, renal biopsy remains mandatory for the definitive diagnosis of IMN and assessment of disease severity.

## References

[1] Ardalan M, Ghafari A, Hamzavi F, Nasri H, Baradaran B, Majidi J, Nikbin B (2013). Anti-phospholipase A2 receptor antibody in idiopathic membranous nephropathy: a report from Iranian population. J Nephropathol.

[2] E. Honkanen, T. Tornroth, C Gronhagen-Riska (1992). Natural history, clinical course and morphological evolution of membranous nephropathy. Nephrol Dial Transplant.

[3] F Ferrario, M.P. Rastaldi (2004). Histopathological atlas of renal diseases: membranous glomerulonephritis. J Nephrol.

[4] Jones DB (1957). Nephrotic glomerulonephritis. Am J Pathol.

[5] Beck LH Jr, Bonegio RG, Lambeau G (2009). M-type phospholipase A2 receptor as target antigen in idiopathic membranous nephropathy. N Engl J Med.

[6] Beck LH Jr, Fervenza FC, Beck DM (2011). Rituximab-induced depletion of anti-PLA2R autoantibodies predicts response in membranous nephropathy. J Am Soc Nephrol.

[7] Kanigicherla D, Gummadova J, McKenzie EA (2013). Anti-PLA2R antibodies measured by ELISA predict long-term outcome in a prevalent population of patients with idiopathic membranous nephropathy. Kidney Int.

[8] Cravedi P, Ruggenenti P, Remuzzi G (2011). Circulating anti-PLA2R autoantibodies to monitor immunological activity in membranous nephropathy. J Am Soc Nephrol.

[9] Qin W, Beck LH Jr, Zeng C (2011). Anti-phospholipase A2 receptor antibody in membranous nephropathy. J Am Soc Nephrol.

[10] Oh YJ, Yang SH, Kim DK, Kang SW, Kim YS (2013). Autoantibodies against phospholipase A2 receptor in Korean patients with membranous nephropathy. PLoS One.

[11] Hoxha E, Harendza S, Zahner G (2011). An immunofluorescence test for phospholipase-A₂-receptor antibodies and its clinical usefulness in patients with membranous glomerulonephritis. Nephrol Dial Transplant.

[12] Hoxha E, Thiele I, Zahner G, Panzer U, Harendza S, Stahl RA (2014). Phospholipase A2 receptor autoantibodies and clinical outcome in patients with primary membranous nephropathy. J Am Soc Nephrol.

[13] Timmermans SA, Damoiseaux JG, Heerings-Rewinkel PT (2014). Evaluation of anti-PLA2R1 as measured by a novel ELISA in patients with idiopathic membranous nephropathy: a cohort study. Am J Clin Pathol.

[14] Glassock RJ (2014). Antiphospholipase A2 receptor autoantibody guided diagnosis and treatment of membranous nephropathy: a new personalized medical approach. Clin J Am Soc Nephrol.

[15] Hoxha E, Kneißler U, Stege G (2012). Enhanced expression of the M-type phospholipase A2 receptor in glomeruli correlates with serum receptor antibodies in primary membranous nephropathy. Kidney Int.

[16] Larsen CP, Messias NC, Silva FG, Messias E, Walker PD (2013). Determination of primary versus secondary membranous glomerulopathy utilizing phospholipase A2 receptor staining in renal biopsies. Mod Pathol.

[17] Hanley JA, McNeil BJ (1982). The meaning and use of the area under a receiver operating characteristic (ROC) curve. Radiology.

